# Max Power: Implementing the Capabilities Approach to Identify Thresholds and Ceilings in Energy Justice

**DOI:** 10.1007/s11948-021-00353-2

**Published:** 2022-02-08

**Authors:** Patrik Baard, Anders Melin

**Affiliations:** Department of Global Political Studies, Malmö University, 205 06, Malmö, Sweden

**Keywords:** Energy justice, Capabilites approach, Capability thresholds, Capability ceilings

## Abstract

In this paper, we apply the capabilities approach—with the addition of capability ceilings—to energy justice. We argue that, to ensure energy justice, energy policies and scenarios should consider enabling not only minimal capability thresholds but also maximum capability ceilings. It is permissible, perhaps even morally required, to limit the capabilities of those above the threshold if it is necessary for enabling those below the threshold to reach the level required by justice. We make a distinction between tragic and non-tragic conflicts of capabilities: tragic conflicts are instances when one cannot raise an agent’s capabilities above the threshold that justice requires without pushing someone else below the threshold or restricting someone from reaching the threshold. In contrast, a non-tragic choice is when increasing someone above the threshold required by justice does not entail pushing someone else’s capabilities below the threshold. We utilise this framework to discuss energy justice and emissions of greenhouse gases. Drawing on the relation between points on the human development index and levels of energy consumption, we conclude that non-tragic mitigation policies now are highly preferable to tragic policies later.

## Introduction

Energy is a central component of welfare, well-being, and development (Arto et al., 2016; Smil, 2008). Per capita energy consumption can be linked not only to human development but also to infant mortality and female life expectancy (Smil, 2008: 347; Steinberger, 2016). While a substantial number of people live in ‘energy poverty’, some nations have substantially high levels of per capita energy footprints. Additional use of energy would do little to raise the well-being of those already well off relative to those that are lesser well-off, whose well-being increases substantially even with modest increases in energy use (Smil, 2008; Steinberger, 2016). Conversely, restricting the energy use of the most well-off would not entail substantial decreases in their well-being.

We will implement the *capabilities approach* (hereafter CA) to energy justice, but suggest that capabilities should be supplemented by capability ceilings in this context. Section 2 surveys different aspects of energy justice, primarily the relation between well-being and energy consumption. Section 3 presents the CA, including both thresholds and ceilings. This is followed by an application of the framework to energy justice and a short summary in Sects. 4 and 5, respectively. After defining capability thresholds and ceilings we define the distance between thresholds and ceilings. We argue that the distance between the threshold and the ceiling for each generation diminishes as the concentration of greenhouse gases increases. This means that a non-tragic choice now shades into a tragic choice later. The CA, supplemented with ceilings, will be proposed as a reasonable theory for energy justice and defining what energy justice requires in practice.

## Energy Justice: Energy Consumption and Well-Being

As a part of formulating a capabilities framework suitable for energy justice, a possible starting point is to discuss the relation between satisfying the criteria of the human development index (HDI) and energy consumption per capita (Arto et al., 2016; Smil, 2008; Steinberger, 2016). Globally, energy consumption often requires burning of fossil fuels. Consequently, increasing energy consumption increases the risks of dangerous climate change. Yet, to not increase energy consumption given current energy technologies means that some parts of the world are not given the chance for economic development and increased well-being, lest highly substantial mitigation is done in high-emitting countries. This leads to energy consumption being a form of ‘good’ that has to be fairly distributed.

An energy consumption of 0–100 Gigajoule (GJ) per capita corresponds to a steep growth in HDI from around 0.3 to 0.8 (Steinberger, 2016; see also Smil, 2008: 347). After 100–110 GJ per capita, the increases in HDI points peter out, and additional energy consumption provides minimal to no gains in HDI (Smil, 2008: 348). For example, countries with as high per capita consumption as 300 GJ or above are approximately at HDI 0.9 (Smil, 2008: 348; see also Steinberger, 2016). Thus, energy consumption on a global level has the shape of a diminishing marginal utility; that is, small increases in energy consumption have a substantial impact on well-being for the least well-off (those consuming under 100 GJ per capita and being below HDI 0.8), whereas substantial increases in GJ per capita are required for minuscule, if any, increases in HDI for those above 100 GJ per capita.

Based on data from the energy company BP, Table 1 shows how the average gigajoule (GJ) per capita consumption in 2018 was distributed across continents.

**Table 1:** Average primary energy consumption (GJ/capita) in 2018 divided by continent^a^. *Source*: BP (2020)

	Africa	South and Central America	Asia Pacific	Europe	Middle east	Commonwealth of independent states	North America
I: GJ per capita	15	56	60	127	148	161	240

^a^Limited to commercially-traded fuels (BP, 2020)

The aggregated data overshadows the great differences between nations’ per capita energy consumption. For instance, Pacific Asia includes Singapore, which consumed 633 GJ per capita that year, and Bangladesh, which consumed only 9 GJ per capita. Moreover, the use of traditional biofuels—such as woody phytomass, charcoal, and crop residue—is very difficult to estimate compared to crude oil, for which there is detailed information about virtually all that enters the market (Smil, 2017: 78). However, traditional biofuels are a common source of energy in low-income countries. Roughly 2.5 billion people are estimated to rely on such energy sources for cooking and heating (Smil, 2017: 87). In contrast, roughly 15% of the world’s population live in high-income economies and claim nearly 45% of all commercial energy (Smil, 2017: 234).

Despite the relation between HDI and energy consumption per capita, there are some concerns about using it to guide claims of justice. Chiefly, there is no agreement on whether energy justice should be assessed with regard to total primary energy *demand* per capita or total primary energy *footprint* per capita (Arto et al., 2016). Measuring only domestic energy demand may limit the accuracy of how much energy that is consumed, and its impacts. Measuring the energy footprint reveals a higher consumption of GJ per capita relative to HDI than domestic energy demand does (Arto et al., 2016: 9). Energy footprints include consumption patterns and energy embedded in products produced in nations other than where they are consumed.

Using the HDI to guide policy goals may also contradict intuitions of justice. Consider the HDI component ‘life expectancy at birth’. HDI will give equal points to the following two expectancies of longevity (Pogge, 2002: 213):

**Table:** 

	Group 1	Group 2
Scenario A	Wealthier half 70 years	Poorer half 30 years
Scenario B	Wealthier half 50 years	Poorer half 50 years

Given that it is easier and cheaper to raise the low life expectancy of younger persons, resources will be devoted to group 2 in scenario A. This choice is consistent with most moral intuitions. However, because greater improvements in the HDI would result from focusing resources on the healthier individuals whose life expectancy can be extended more cheaply, through access to ‘the most basic nutrition, sanitation, and medical care’, use of the HDI to determine what energy justice requires would lead to withholding of ‘scarce resources from those who have special needs that make their life expectancy more expensive to extend’ (Pogge, 2002: 213).

The relation between energy consumption and fossil fuels poses a risk that energy justice solely concerns mitigation. Surely, principles for establishing what is a fair quota of emissions (Caney, 2005), or what entails luxury emissions and subsistence emissions (Shue, 2014 [1993]), are investigated in climate justice, which includes principles based on historical emissions (such as polluter pays-principle, or beneficiary pays-principle), or current abilities (ability to pay-principle). Yet, we will limit the discussion to the relation between HDI and energy use, interpreted through the lens of CA. We hope to show that when supplemented with ceilings, CA is instructive as it ties the discussion to the capabilities that energy use must be relevant for.

Although there are similarities energy justice has a wider scope than climate justice. Energy justice is not limited to mitigation but includes concerns such as the environmental impacts of renewables, technological issues such as developing smart grids and off-grid energy technologies, and topics such as the relation between property rights and construction of renewables and development of novel technologies (see van der Graaf & Sovacool, 2020). Energy justice is not limited to distributive justice, but also include procedural justice, and recognition (Sovacool, 2013). Whether renewable energy sources can develop at a fast enough pace to provide a sufficient share of the global energy to enable a just transition is disputed (Smil, 2019). A premise here is that renewables will provide a modest share of the global energy mix, and consequently, lowering emissions will often entail lowering energy use. But we will also consider the development of renewables and increased energy efficiency as possibilities for maintaining high levels of energy use per capita. We aim to show that the CA supplemented with ceilings is a reasonable theory to be applied to energy justice and establishing criteria for dividing burdens and benefits of just transitions.

## Specifying Thresholds and Ceilings and Their Relation

In this section, we present the CA supplemented with capability ceilings. We also discuss how the standard CA threshold relates to the ceiling. This provides the framework for our discussion of energy justice in the subsequent section.

### The Capability Approach

Human capabilities, as a way of defining well-being, are helpful for reasoning about what energy justice requires. Capabilities define what opportunities a person has to do or be something, and not what a person has. In contrast, *functionings* are what a person actually does or is, such as being a parent or working as a doctor. Having capabilities presupposes having the freedom to choose one’s functionings. For both Nussbaum and Sen, freedom of choice is essential for a good human life (Nussbaum, 2006; Sen, 1999). A central issue of CA related to energy is to make people equally capable, not give them an equal amount of energy. Given the relation between energy usage and well-being, energy access is a prerequisite for many capabilities, and the CA is instructive regarding *what* energy availability ought to enable.

The CA has many benefits. A common starting-point when discussing the currency of justice (Page, 2007) that can serve as a contrast to CA is welfarism, being a view that ‘restricts the judgments of state of affairs to the utilities in the respective states (paying no direct attention to such things as the fulfillment or violation of rights, duties, and so on)’ (Sen, 1999: 59). That is, the aggregate level of utility is given precedence in welfarist theories of distributive justice, and ‘injustice’ is restricted to ‘aggregate loss of utility compared with what could have been achieved’ (Sen, 1999: 59). An unjust society is one which consists of less level of utility, measured by for instance happiness or preference satisfaction, than it could have consisted of. The aggregative nature of welfarism suffers from distributional indifference and a neglect of rights, freedoms and concerns that cannot be translated to utilities (Sen, 1999: 62). Moreover, welfarism risks ignoring preferences that are adapted to detrimental conditions, thus fulfilled already under relatively harsh circumstances (Sen, 1999: 62). In contrast, CA focus on ‘people’s substantive freedom to achieve the life that they have reasons to value’, being ‘human ‘functionings’ and the ‘capabilities’ to achieve them’ (Page, 2007: 461; Sen, 1999). CA is less vulnerable to the accusations to welfarist accounts, since CA is not as restricted to aggregate preference satisfaction. Rather, some CA theorists, such as Martha Nussbaum, identifies a list of ten capabilities as prerequisites of leading a decent life (Nussbaum, 2006). Others, such as Amartya Sen, does not provide a set list, but rather focus on the relation between capabilities as freedom, health, and life (Sen, 1999). Sen argues for a contextual version of CA, according to which the capabilities should be determined through public reasoning and open debates (Sen, 1999).

Capabilities denote core human entitlements ‘that should be respected and implemented by the governments of all nations, as a bare minimum of what respect for human dignity requires’ (Nussbaum, 2006: 70). The CA allows a wide enough applicability that adheres to multiple theories of human nature and accommodates the value pluralism of liberal societies (Nussbaum, 2006: 70). Capabilities ought to be abstract enough ‘to leave room for the activities of specifying and deliberating by citizens and their legislatures and courts’ but specific enough to offer substantive content (Nussbaum, 2006: 78ff). Thus, the CA sets a minimal threshold of capability levels that all individuals are entitled to, being a minimum of what is required to live a life worthy of human dignity.

To Nussbaum (2006), the threshold level is a level ‘beneath which it is held that human functioning is not available to citizens’ (71). The CA has several practical repercussions to policy. First, an aggregated level is not adequate for assessing whether a decision is right, as a loss of someone’s capabilities cannot be ‘compensated’ by raising someone else’s. Second, a government that does not ensure that all the capabilities can be met performs an injustice on its citizens. That is, if just one capability is not ensured, a government commits a fault of basic justice (Nussbaum, 2006: 167). Capabilities are to be understood as minimal entitlements for all individuals by virtue of being humans and having dignity and sociability; accordingly, they have minimal thresholds that no one should fall under.

### Capability Ceilings

Nussbaum admits that the CA does not provide a complete account of social justice and that it is ‘an account of minimum core social entitlements’ (Nussbaum, 2006: 75). Holland (2008, 2014) has developed a framework that supplements capability thresholds with ceilings. Capability ceilings ‘establish maximum levels on the protection and provision of capabilities that can cause harm to others’ (Holland, 2014: 142); namely, they ‘impose a limit on the set of basic opportunities available to people’ (Holland, 2008). Capability ceilings establish maximum levels and are thus a supplement to capability thresholds, which establish the minimum levels. Accordingly, ceilings impose restrictions as they ‘make people unable to do certain things’ (Holland, 2014: 142). A ceiling on capabilities also means a ceiling on functionings, as it constrains what functionings someone can achieve. Because CA emphasizes having freedom of choice, a ceiling on capabilities rather than on functionings is more in line with the approach as a whole.

Capability ceilings are required ‘to resolve capability conflicts by securing the central human capabilities at a threshold level for everyone’ (Holland, 2014: 143). The concept of ceilings, understood as limits to other’s actions, is somewhat similar to other principles such as John Stuart Mill’s ‘harm principle’, which justifies governmental restrictions on the freedom and actions of individuals to prevent harm to others (Mill, 1956; Holland, 2014: 145). Thus, the CA is compatible with at least some liberal principles of justice.

Capability ceilings are akin to other suggestions. For instance, versions of *sufficientarianism* includes upper limits beyond which further distribution is not required (Crisp, 2003). That is, including the entitlements or claims of an agent, B, is only appropriate ‘up to the point at which B has a level of welfare such that B can live a life which is sufficiently good’ (Crisp, 2003: 762). This version of sufficientarianism is however limited to the claims of an agent playing a reduced role when determining what is required by justice once that agent is above a level recommended by sufficientarianism. In contrast, and more similar to capability ceilings, *limitarianism* recommends a firm upper level, beyond which any surplus not required for a fully flourishing life is to be devoted to increasing the well-being of others (Robeyns, 2019).

Thus, *capability ceilings* are maximum limits—or constraints—to capabilities. As a more concrete example, Holland discusses driving SUVs as an instantiation of the capability for bodily integrity, which includes ‘being able to move freely from place to place’ (Holland, 2014: 147). Most governments provide alternative transportation opportunities for people who cannot afford SUVs in the form of, for instance, public transport. However, the SUV owner can move more freely than those restricted to public transport. Applying the capability ceiling concept, a government can provide different incentives that redirect resources in a manner ‘that will secure threshold levels of capabilities for people who experience mobility conditions that are below the threshold level that justice requires’ (Holland, 2014: 148). Practically, this could mean, for instance, imposing a SUV tax, the revenues of which ‘would go toward improving the availability of public transportation for those who currently lack access to basic means of mobility’ (Holland, 2014: 148).

There are various justifications for a CA that is supplemented with ceilings. First, one can understand ceilings as limitations on use of luxury items (Holland, 2008: 417). That they are *luxury* items means that they are contributing to capabilities that are already well above the threshold that justice requires securing for each person. Forfeiting them, or taxing them or limiting their use, would not entail a morally relevant sacrifice if it does not push the agent utilising them below the threshold. This is similar to Henry Shue’s distinction between luxury and subsistence emissions (2014 [1993]), while luxury emissions are not morally relevant subsistence emissions are. Yet, he leaves it quite open as to where the difference is (Shue 2014 [1993]: 66). The CA applied to energy is instructive given that it establishes what energy availability should enable. Second, celings would, as suggested above, divert resources that could go to more fundamental capability protections (Holland, 2008: 417).

Capability ceilings are warranted when conflicts arise. Such conflicts come in two forms: tragic and non-tragic. A capability conflict is tragic when the following condition is fulfilled:

When it is *impossible* to push the capabilities of one person to the threshold level that justice requires without simultaneously pushing the capabilities of another person below the threshold level that justice requires. (Holland, 2014: 136)

In contrast, a non-tragic conflict entails a limit or restriction on the enjoyment of one agent’s or group of agents’ capabilities that would not push them below the threshold.

Consider the SUV case above. If the SUV driver ceases ‘pleasure driving’ in an SUV, it would free up money spent on gasoline, but there would also be health gains (Holland, 2008: 417; Nussbaum, 2006: 403) and omitted gas emissions. Moreover, some would claim that pleasure-driving an SUV is not a morally neutral act since it exposes others to harm (Hiller, 2011; Nolt, 2016). However, imposing such a restriction would limit an agent’s mobility and potentially infringe on property rights, or at the very least on the use of property. This reduces the two capabilities of bodily integrity (which includes being able to move freely) and control over one’s environment (which includes property rights) (Holland, 2008: 417). Such restrictions are, in this framework, motivated when required to allow for others to reach a capability threshold. It could be in the form of a fair sharing of a common good being a prerequisite for capabilities, or imposing taxation.

While the existence of ceilings may be consistent with intuitions of justice when there are great asymmetries of access to and consumption of goods required for subsistence, an additional concern is whether the ceiling is equivalent, or not, to the threshold.

### The Ceiling Relative to the Threshold and the Issue of Priority

If we assume that a CA supplemented with ceilings is reasonable, how much is one permitted or required to restrict agents’ capabilities when they have reached the threshold and others are below it? If the capability ceiling is equivalent to the threshold, then a restriction applies to an agent just after it has reached the threshold. While Holland concedes that the threshold and ceiling overlapping may seem reasonable, she also writes that ‘the capability to control one’s material environment might allow for a ceiling that is set far above the threshold’ (Holland, 2008: 419). For instance, a property owner may extract groundwater from their property above a level required for subsistence, for a variety of other reasons, but only up to the point at which further extraction ‘starts to undermine other central capabilities’ (Holland, 2008: 419). Consequently, we may specify *the threshold–ceiling relation* in the following manner:

The threshold and the ceiling are *equivalent* if passing the threshold results in others not reaching up to the threshold. The threshold and the ceiling are *not equivalent* if passing the threshold is consistent with others reaching up to the threshold.

Restricting capabilities of those that have reached high levels above the threshold is required within the CA when supplemented with ceilings. The requirement of such ceilings is motivated when they are necessary for allowing others to reach the threshold. Giving the capabilities of those under the minimal threshold levels absolute priority is consistent with setting the ceiling equivalent to the threshold, *if* that is required. When capabilities require consumption of finite resources, it may very well be the case that the threshold and ceiling are equivalent suggesting that no one is entitled to consume *more* than is necessary for that capability, as doing so would mean blocking others from reaching their capability thresholds. This is what the example of the groundwater illustrates. What such a requirement results in may differ regarding the means necessary to fulfill specific capabilities and the capabilities that one is considering.

When the imposition of a capability ceiling does not entail pushing people below the threshold to allow others above it, it is a non-tragic choice. A different picture emerges if there are tragic choices (Holland, 2014: 136; Holland, 2008: 415). Such conflicts require paying heed to the tragic question of whose capabilities will be sacrificed (Holland, 2014: 136). To Nussbaum, such conflicts are ‘a sign that society has probably gone wrong somewhere; that it is probably not well designed’ (Nussbaum, 2006: 401). Furthermore, they ‘initiate long-term planning efforts that will allocate resources away from supporting entitlements that are not fundamental and toward supporting threshold level of capabilities that define the conditions of justice’ (Holland, 2008: 415).

There are possibilities for managing tragic choices. For instance, one can potentially lower the threshold to such a level that more can reach it. However, this would be a curious suggestion given that capabilities ought to be consistent with the type of ‘being’ they refer to and the value and needs of that ‘being’. It would be as if one is justifying *less* dignity to persons. However, even if we assume that the threshold level should reflect the type of ‘being’ that humans are, and their moral worth and sociability, there is some flexibility in where to set the threshold (Nussbaum, 2006: 403). While it may be obvious that some activities, such as pleasure-driving SUVs, are well beyond thresholds (Nussbaum, 2006: 403), it is not immediately clear where to set the threshold.

Another possibility when there are finite resources is that the threshold remains but that an equal distribution result in all agents being under the threshold. For example, consider the sharing of a resource that generates 100 units of something. What is needed for minimal level of capabilities is 25, and there are five agents. Assuming that there are no morally relevant differences between the five agents that would warrant an unequal distribution, there are (at least) two main distributive options consistent with justice:

**Table:** 

	1	2	3	4	5
A	20	20	20	20	20
B	25	25	25	25	0

Choosing A is consistent with egalitarian and prioritarian principles (Parfit, 2002). The CA is somewhat uninstructive when it is not possible to ensure that *all* can reach the threshold. It fails to provide guidance on whether it is better to permit one person below the threshold to allow others to reach it, which would result in an unequal distribution of a common resource, or to keep all equally bad-off under the threshold. Given that not all agents have reached the threshold in A and B, the CA seems, counter-intuitively, to fail to provide guidance between them. It would be possible that the CA would recommend choice B, but recall that Nussbaum insisted that if even one core capability is not ensured, a government commits a fault of basic justice (Nussbaum, 2006: 167). Yet, the CA would be consistent with B if we assume that the CA would recommend maximizing the number of people who are above the threshold, despite one agent being well below it. Ruling that A is better would require introducing additional principles to CA, such as forms of egalitarianism or prioritarianism. Despite such issues, it seems reasonable that ceilings are justified when a common good is a prerequisite for capabilities.

## Energy Justice and the Supplemented Capability Framework: Avoiding Tragic Choices

Few have considered the application of capability ceilings in energy justice. A few things are required for doing so. First, we must investigate whether the HDI can be reasonably translated to capabilities. Second, we must consider ways of non-arbitrarily establishing a threshold level for HDI (and capabilities). Third, and similarly to the second point, we must find a way to non-arbitrarily establish a ceiling for energy concumption per capita in relation to the HDI points.

The HDI has a significantly narrower scope than the capabilities listed by Nussbaum, but is more consistent with the capability approach as discussed by Sen. Recall that Sen discussed the capability approach as providing opportunities substantial to achieve freedom (1999). Energy consumption per capita relates to infant mortality, female life expectancy, and political freedom, in addition to HDI (Smil, 2008: 347), which all affects agents’ capabilities. While the relation between energy use and HDI most likely requires taking other components into consideration, for the sake of simplicity we will limit the discussion to energy use as a prerequisite for capabilities.

Indicators of HDI are life expectancy at birth, expected and mean years of schooling, and gross national income (GNI) per capita (UNDP, 2020). Life expectancy quite neatly translates to the capability ‘Life’, defined as ‘being able to live to the end of a human life of normal length’ or ‘not dying prematurely’ (Nussbaum, 2006: 76). While not equivalent to expected and mean years of schooling, education is included in the capability ‘Senses, imagination, and thought’, which stresses the ability to use these faculties in ‘a way informed and cultivated by an adequate education, including, but by no means limited to, literacy and basic mathematical and scientific training’ (Nussbaum, 2006: 76). The category ‘a decent standard of living’ is reduced to a single economic measure. Such a measure has no obvious correlation with the capabilities but may refer to the agency and capacity to choose that permeates and motivates the capabilities. However, this means that there are many of the capabilities that Nussbaum mentions that are not included in the HDI, which may speak to the limitation of the HDI rather than be a limitation of the CA. Despite the above, the HDI could be helpful for understanding the relationship between per capita energy consumption and at least some of the dimensions of human well-being that are necessary for living a life worthy of human dignity. Strengthening the relation between HDI and capabilities is how HDI primarily was originated by Sen (Gasper, 2002: 444). Sen focus on the CA as prerequisites for functionings, the choices that people make, and HDI is meant as an indicator for a good life (Gasper, 2002: 444).

Both HDI and CA have the strengths of avoiding many of the problems associated with other theories concerning the currency of justice, such as welfarist accounts that was described above. Being based on preference satisfaction, welfarist accounts need not give precedence to those that are below a certain level, for several reasons. First, it may theoretically be the case that the preferences of those below the threshold are satisfied, due to those preferences being adapted to what is realistically achievable in harsh conditions (see Page, 2007: 455). Second, those above the threshold may be less well-off than they could be, which would justify increasing their already substantial consumption of energy. This is a variation of the ‘expensive tastes’ problem (Crisp, 2003) and of ‘luxury emissions’ (Shue, 2014 [1993]). Given that the relation between energy consumption and HDI has the shape of a diminishing marginal utility, it is likely that this would require substantial increases in energy consumption to increase preference satisfaction. This is similar to satisfying the preferences of someone who has cultivated expensive tastes in food and wine. In contrast, which adds to its reasonableness as a theory of justice, the CA would restrict its focus to those that are below the threshold, and when supplemented with ceilings would exclude the moral relevance of ‘luxury’ preferences and when satisfaction would be at the expense of those below the threshold. In contrast, preferences are adaptive to poor conditions, and focusing too narrowly on preference satisfaction may be inconsistent with the demands of justice (Nussbaum, 2006; Sen, 1999). The CA is more consistent with fulfilling the demands of justice in this regard, as it avoids the problem of adaptive and aggregative preferences.

### Capability Thresholds and Ceilings in Energy Justice

A proposal for how to include thresholds in energy consumption is provided in Scenario II of Table 2.

**Table 2:** Distribution of GJ per capita over different agents, between continents (Scenario I) ( Source: BP 2019), and by increasing the GJ per capita of one agent, C, (Scenario II)

Scenario	A	B	C	D	E	F	G
I (current): GJ per capita	15	56	60	127	148	161	240
II (cap approach): GJ per capita	15	56	100	127	148	161	240

Since we know approximately the points at which the energy consumption per capita reflects different HDI points, converting HDI to capabilities means that we would also know the points at which energy consumption per capita to secure the minimum thresholds of some capabilies. For instance, if we assume that an HDI of 0.8 (labelled ‘Very high’ by the UN), which requires roughly 100 GJ per capita, is an adequate proxy for the capabilities relevant for HDI, then, as a matter of global justice, levels should be raised for A, B, and C being entitled to increased energy availability. Raising C, rather than A or B, is consistent with Pogge’s criticism of the incentives that HDI provides (2002). Given that the GJ per capita is raised for C, Scenario II would be a distribution more consistent with the CA, assuming that the HDI of C is also raised.

But the CA can provide more guidance than merely identifying Scenario II as consistent with justice. Considering that coal, oil, and gas made up approximately 80 per cent of the global primary energy sources in 2017 (Valentine et al., 2019: 2), raising the energy availability for A, B, or C would simultaneously entail increasing the burning of fossil fuels. In turn, this would increase the risk of dangerous climate change for future generations. If energy use is a prerequisite for capabilities, those that consume too much are committing a wrong *if* their usage limit the energy use required for others’ capabilities thresholds. If energy use does not limit others reaching above the threshold, then ceilings are not motivated. Applying the capabilities ceiling, it would not be beyond the scope of justice to limit energy usage of those consuming high levels, where the limitation does not push any agent below the thresholds of entitlements; rather, it is a non-tragic choice. Such limitations are also found elsewhere in climate justice, such as Caney’s suggestion of a fair quota and a duty to not breach that quota, and a right to emit for the least advantaged (2005), or Shue’s discussion on luxury emissions (2014 [1993]).

Even if C can be raised to 100 (assuming that it would suffice for reaching the threshold), it is uncertain where those additional 40 GJ per capita would come from relative to scenario I. They could be derived from a redistribution of fossil fuels or an intensified search for new resources; however, if that were the case, it would increase greenhouse gas emissions, which in turn would increase the risk for dangerous climate change. Alternatively, the increase for C could be enabled by renewable sources—either in the form of C utilising renewable sources or D–G forfeiting their use of fossil fuels for C’s benefit and covering the loss with renewables to maintain their levels of average GJ per capita. This seems unlikely given the modest efficiency of renewables and their scarce share in the sources of total energy consumption (Smil, 2017; Valentine et al., 2019). If no technological leaps quickly occur that make renewables highly efficient, the distribution will most likely be of fossil fuels. According to some the development and global distribution of renewables is unlikely to be anything but a decadal long strategy (Smil, 2017).

### Capability Ceilings and Global Energy Reductions

Increasing usage of fossil fuels would raise the risk of dangerous climate change and consequently lower the capabilities of later generations. Therefore, abating such a risk entails a redistribution that does not increase the total amount of greenhouse gas emissions. This would generate the distribution of Table 3 to get A–C above the threshold without pushing D–G below it while still requiring that D–G make noteworthy reductions (i.e. a non-tragic choice):

**Table 3:** Distribution of GJ per capita consistent with CA supplemented with ceilings

Scenario	A	B	C	D	E	F	G
III (cap. threshold and ceiling): GJ per capita	100	100	100	117	120	130	140

Scenario III is of course overly simplified, as aspects such as energy efficiency and the level of useful energy will be factors when measuring HDI relative energy consumpsion. Scenario III is effectively putting a ceiling on the capabilities of D–G. There are likely technical difficulties that prevent any clear judgment regarding distribution. For instance, could energy forfeited by G be transferred to A in any feasible manner, and will it be as efficient when turned into useful energy by A? This will depend on the types of energy. Fossil fuels have the merits of being easily redistributed and utilised—preferably utilised in as highly efficient manner as possible and without causing ill health and danger (which is common in, for instance, traditional kitchen stoves) (Smil, 2008: 349). There are currently modest possibilities for meeting increased energy consumption on a global level without increasing emission of fossil fuels, but alternatives in developing countries would include ‘off-grid’ electricity, and possible ‘leapfrogging’ energy stages (van de Graaf & Sovacool, 2020: 71) to meet growing energy demands without increasing emission levels, conjunct with ambitious mitigation policies of high-emitters.

However, it would make sense to raise A–C to an appropriate level of HDI (and thus sufficient levels of some capabilities) by increasing energy availability without E, F, or G making morally relevant sacrifices (lowering the levels of their energy demand without morally relevant decrease in HDI or capabilities). Nussbaum considers an example that is similar by way of principle when she writes, ‘There are very likely other costs not associated with fundamental entitlements that could be trimmed way back before we would have to cut anyone’s health care […] surely support for luxury items would be our first target’ (Nussbaum, 2006: 403).

Nevertheless, scenario III is problematic if it allows maintaining current levels of greenhouse gas emissions. According to most estimates, current levels are well beyond safe boundaries. During the period 1970 to 2015, the global primary energy consumption increased from 223.35 EJ to 525.49 EJ the majority of which comes from fossil fuels (Smil, 2017: 240ff). The lack of action from one generation will result in required intensified efforts by subsequent generations to limit the effects of dangerous climate change. Many could reduce their carbon footprint without being pushed below the threshold level while still allowing the least well-off to increase their emissions, and thus their HDI and by extension (some of) their capabilities, being a non-tragic choice. Doing so would make it possible to reduce dangerous climate change in addition to enabling the least well-off of that generation to increase their emissions and well-being. Given that the relation between HDI and energy consumption is that of a diminishing marginal utility, very small increases in energy consumption for the least well-off would result in significant increases in HDI.

Choosing not to lower total emissions becomes an increasingly tragic choice to posterior generations facing much more aggressive mitigation efforts *or* increasing the likelihood and magnitude of dangerous climate change. Either way, the risk of pushing individuals, either current or future, below the threshold of capabilities becomes more severe. If a first generation in a sequence of generations expected to mitigate does not reduce emissions, it will force the immediately subsequent generation to significantly reduce their emissions (Gardiner, 2011) and possibly to accept that not all from that generation will reach the threshold required by justice. The tragedy of that policy would be that it pushes people below the threshold, as increasingly aggressive mitigation policies would be required. Alternatively, that second generation could behave as the preceding generation. This will mean that the third generation will face an even more severe challenge of picking up the slack for two generations. Eventually, dangerous climate change will set in, which will most likely substantially increase the number of people who are pushed below the threshold for many more capabilities than those covered by the HDI and relevant to energy justice.

For each generation, the distance between the ceiling and the threshold diminishes, and with it, non-tragic choices shade into tragic choices. For each generation, there is, globally, the choice of either aggressively mitigating GHG or exposing future generations to dangerous climate change. For each generation, it would be better for those that can reduce their carbon footprint without being pushed below the threshold to do so—a non-tragic choice—in order to both reduce the risk of future dangerous climate change and allow peers of that generation to increase their capabilities to the threshold.

### Options for Avoiding Tragic Choices

One possible way of avoiding the tragic choice while simultaneously ensuring justice would be to swiftly develop energy technology that can produce energy to meet current and increasing levels of demand without increasing the risks of dangerous climate change, which is likely a decadal long affair (Smil, 2017). Another possibility is to investigate technical measures such as carbon capture and storage (CCS) or direct air capture technologies (Preston, 2018). However, while there are no shortages of CCS being included in plans, if all the CCS projects that were planned for 2015 had been built, it would have sequestered approximately 0.2 per cent of the total emissions in 2015 (Smil, 2017: 184). Moreover, such novel technologies are untested on the required scale, and negative environmental effects are possible.

Another choice is to restrict emissions of greenhouse gases. Viewed inter-generationally, the CA supplemented with ceilings recommends such a choice. Since energy demands are primarily fulfilled through the burning of fossil fuels, then both avoiding climate change and ensuring (or at the very least raising the likelihood) that future generations get what is required by justice would entail substantial restrictions on current and near-term emissions of fossil fuels. Given the close relation between HDI and energy use per capita, this likely means that a great share of the current generations are not provided what is required by justice. This would be a tragic choice wherein future generations can be pushed up to the threshold at the expense of members of current generations.

However, there are some strategies to get out of this tragic choice. First, one may not regard all of posterity when discussing the inter-generational aspect. Rather, one can assume that the capabilities either apply only to the people who are currently existing or apply to the immediately subsequent generations. If *all* of posterity is included, that requires considering the capabilities of a set of potentially infinite persons, or at the very least a very high amount. On the other hand, it may be counter-intuitive to restrict capabilities only to those currently existing when the consequences of one’s choices will not be limited to currently existing individuals. If that were the case, then the storage of nuclear waste, as an example of decisions with long-term consequences, would provide little moral problem; it will most likely affect those existing in the far-off future, not anyone currently existing. Nevertheless, it seems morally questionable to reach such a conclusion.

In contrast, one may limit the concern to capabilities of the immediately subsequent generations. They are likely to require the same or similar capabilities as us, through similar foreseeable means, and our decisions will have an immediate impact on their capabilities. This may suffice, but it would still entail large sacrifices when it comes to mitigating emissions. However, a large portion of those sacrifices would not be morally relevant, as it would involve ‘luxury emissions’. If lowering such emissions does not require pushing people below the threshold, it is a non-tragic choice. Stated differently, policies to *lower* the GJ per capita emissions and energy use of those above the threshold, to permit for others below the threshold to reach above it is consistent with the CA supplemented with ceilings.

Alternatively, one can reiterate the view proposed by Nussbaum that tragic conflicts are a sign that ‘society has probably gone wrong somewhere’ (Nussbaum, 2006: 401). To Holland, such conflicts should ‘initiate long-term planning efforts that will allocate resources away from supporting entitlements that are not fundamental and toward supporting threshold level of capabilities that define the conditions of justice’ (Holland, 2008: 415). While it may be possible to still raise everyone to the threshold levels through imposing ceilings and redistribution of resources and ensuring energy availability of the least well-off, resources in the future may still be too scarce to allow everyone to be over the threshold, which would result in tragic choices. It is more consistent with demands of justice to impose non-tragic ceilings now than to enjoy unrestricted freedom and face tragic choices later.

One possible objection is that it could not be required to *lower* the energy use per capita since HDI levels would then decrease, even for high-emitters. For instance, the USA consume roughly 240 GJ per capita, and the EU 127 GJ per capita (see Table 1), both having very high HDI well above 0.8. Possibly, it could not be demanded that these countries lower their GJ per capita, and consequently lower their HDI, since their capabilities are then curtailed.

It is questionable whether this objection holds. It is highly more efficient in HDI terms to allow increased energy use for those at very low rates of GJ per capita (Steinberger, 2016). Moreover, rejecting policies to lower the GJ per capita of those well-off would entail either sacrificing the economic development and increased well-being of poor nations, or allowing such development but at the expense of exposing future generations to increased risk. The fairness of maintaining ‘the accustomed affluence’ of rich nations is highly questionable (Shue, 2014 [1993]: 66). An alternative, suggested in Scenario III, is that high GJ per capita is maintained with significant, possibly unprecedented (Smil, 2019), development of renewable technologies to decarbonize. An alternative, carrying other risks, is the use of nuclear power. For reasons of space we will not further discuss that alternative.

Another potential objection is the challenge of specifying where the ceiling is. If the ceiling is very high then it would only affect the most extreme emitters, largely making capability ceilings irrelevant. It is challenging to pinpoint exactly where to put the ceiling in terms of HDI, a difficulty that is also relevant to other instructive theories such as pinpointing where the difference between subsistence and luxury emissions is (Shue, 2014 [1993]), and how to establish a ‘fair quota’ of emissions (Caney, 2005). But given that a redistribution of energy consumption per capita would increase HDI for the least well-off, that seems like a justified choice. CA supplemented with ceilings would provide room for energy use for the least well-off, currently most likely in the form of fossil fuels but also of off-grid renewables. But it would also restrict the energy use of high-emitters as a matter of justice.

## Summary

In this article, we have shown how the CA supplemented with ceilings can help to think through what energy justice requires in practice, at the level of per capita energy consumption. Such ceilings have the function of restricting agents’ capabilities if required to allow others to reach the thresholds. In some cases, but not all, the ceilings are equivalent to the threshold, meaning that an agent is just entitled to reach (but not pass) the threshold so that others can reach it. Drawing on Holland (2014), we also stipulated tragic and non-tragic choices—the former being instances when it is impossible to push someone above the threshold without simultaneously pushing someone else below the threshold.

We have suggested that such a framework can be used to implement energy justice. First, we discussed that transforming capability thresholds to HDI, which are relevant to some capabilities, reveals a diminishing marginal utility rate between HDI and energy consumption (measured as GJ per capita). This means that even modest increases in energy availability for the least well-off will have substantial impacts on some of their capabilities. Conversely, it means that even substantial reductions in energy use per capita of the most well-off will have a minuscule effect on their well-being Second, we suggested that utilising this framework is instructive when setting ceilings—namely, setting restrictions on energy use per capita—without pushing anyone below the threshold. Third, we argued that the distance between the threshold and the ceiling for each generation diminishes as the concentration of greenhouse gases increases. This means that a non-tragic choice now shades into a tragic choice later, where choices must be made on whose capabilities to sacrifice and by what principles.
